# Early Clinical Features of Dengue Virus Infection in Nicaraguan Children: A Longitudinal Analysis

**DOI:** 10.1371/journal.pntd.0001562

**Published:** 2012-03-06

**Authors:** Hope H. Biswas, Oscar Ortega, Aubree Gordon, Katherine Standish, Angel Balmaseda, Guillermina Kuan, Eva Harris

**Affiliations:** 1 Division of Epidemiology, School of Public Health, University of California, Berkeley, California, United States of America; 2 Sustainable Sciences Institute, Managua, Nicaragua; 3 Division of Infectious Diseases and Vaccinology, School of Public Health, University of California, Berkeley, California, United States of America; 4 National Virology Laboratory, Centro Nacional de Diagnóstico y Referencia, Ministry of Health, Managua, Nicaragua; 5 Sócrates Flores Vivas Health Center, Ministry of Health, Managua, Nicaragua; Universidade de São Paulo, Brazil

## Abstract

**Background:**

Tens of millions of dengue cases and approximately 500,000 life-threatening complications occur annually. New tools are needed to distinguish dengue from other febrile illnesses. In addition, the natural history of pediatric dengue early in illness in a community-based setting has not been well-defined.

**Methods:**

Data from the multi-year, ongoing Pediatric Dengue Cohort Study of approximately 3,800 children aged 2–14 years in Managua, Nicaragua, were used to examine the frequency of clinical signs and symptoms by day of illness and to generate models for the association of signs and symptoms during the early phase of illness and over the entire course of illness with testing dengue-positive. Odds ratios (ORs) and 95% confidence intervals were calculated using generalized estimating equations (GEE) for repeated measures, adjusting for age and gender.

**Results:**

One-fourth of children who tested dengue-positive did not meet the WHO case definition for suspected dengue. The frequency of signs and symptoms varied by day of illness, dengue status, and disease severity. Multivariable GEE models showed increased odds of testing dengue-positive associated with fever, headache, retro-orbital pain, myalgia, arthralgia, rash, petechiae, positive tourniquet test, vomiting, leukopenia, platelets ≤150,000 cells/mL, poor capillary refill, cold extremities and hypotension. Estimated ORs tended to be higher for signs and symptoms over the course of illness compared to the early phase of illness.

**Conclusions:**

Day-by-day analysis of clinical signs and symptoms together with longitudinal statistical analysis showed significant associations with testing dengue-positive and important differences during the early phase of illness compared to the entire course of illness. These findings stress the importance of considering day of illness when developing prediction algorithms for real-time clinical management.

## Author Summary

Dengue virus causes an estimated 50 million dengue cases and approximately 500,000 life-threatening complications annually. New tools are needed to distinguish dengue from other febrile illnesses. In addition, the natural history of pediatric dengue early in illness in a community-based setting has not been well-defined. Here, we describe the clinical spectrum of pediatric dengue over the course of illness in a community setting by using five years of data from an ongoing prospective cohort study of children in Managua, Nicaragua. Day-by-day analysis of clinical signs and symptoms together with longitudinal statistical analysis showed significant associations with testing dengue-positive and important differences during the early phase of illness compared to the entire course of illness. These findings are important for clinical practice since outside of the hospital setting, clinicians may see dengue patients toward the beginning of their illness and utilize that information to decide whether their patient has dengue or another febrile illness. The results of these models should be extended for the development of prediction algorithms to aid clinicians in diagnosing suspected dengue.

## Introduction

Dengue virus (DENV) causes the most prevalent mosquito-borne viral disease affecting humans, with 2.5–3 billion people at risk for infection and approximately 50 million cases of dengue each year [1], [2]. The four DENV serotypes are transmitted to humans by *Aedes aegypti* and *Ae. albopictus* mosquitoes, primarily in urban and peri-urban areas in tropical and subtropical countries worldwide. Most cases present as classic dengue fever (DF), a debilitating but self-limited illness that manifests with high fever, retro-orbital pain, severe myalgia/arthralgia, and rash. However, in some cases, mainly children, illness progresses to life-threatening dengue hemorrhagic fever/dengue shock syndrome (DHF/DSS), characterized by vascular leakage leading to hypovolemic shock and a case fatality rate up to 5% [1], [3], [4]. Currently, no licensed vaccine or antiviral therapy exists for dengue. Early identification of patients at risk of developing severe dengue is critical to provide timely supportive care, which can reduce the risk of mortality to <1% [1], [2]. However, distinguishing dengue from other febrile illnesses (OFIs) early in illness is challenging, since symptoms are non-specific and common to other febrile illnesses such as malaria, leptospirosis, rickettsiosis, and typhoid fever [5]–[7] in dengue-endemic countries. In addition, many distinguishing clinical features of DHF/DSS generally emerge only after 4–5 days, at defervescence, when the patient is already critically ill.

Although the World Health Organization (WHO) has recently established new clinical guidelines to classify dengue severity [1], serological, virological, and molecular biological tests are required to definitively diagnose DENV infection. In many endemic countries, laboratory diagnosis of dengue is often problematic due to lack of reagents, expense, or delay in obtaining results. Patients with suspected dengue are often hospitalized for close monitoring to ensure proper treatment if they begin to develop severe dengue; however, up to 38–52% are later diagnosed with OFIs [8], [9] and thus were hospitalized unnecessarily at great financial cost to their family and society [10]. New tools are therefore needed to distinguish dengue from OFIs to prevent deaths from severe dengue and to mitigate the economic burden of excess hospitalization.

Recent approaches using multivariable logistic or linear regression models have shown that petechiae, thrombocytopenia (platelet count ≤100,000 cells/mm^3^), positive tourniquet test, rash, and other signs and symptoms can distinguish dengue from OFIs [11]–[17]; however, results were not consistent across studies. Only two studies considered clinical and laboratory features according to day of illness [18]–[20], but as these were hospital-based studies, the results likely reflect patients with more severe symptoms and not the clinical spectrum of all symptomatic cases in dengue-endemic populations. Furthermore, none of these studies analyzed data using longitudinal statistical methods, which account for correlations between repeated measures on individuals over time. The use of longitudinal statistical methods to analyze cohort data is essential to utilize all of the data available for analysis and appropriately estimate the within-person and between-person variance in measures over time.

In this study, we used five years of data from an ongoing prospective cohort study of approximately 3,800 children aged 2–14 years in Managua, Nicaragua, to examine the frequency of clinical signs and symptoms by day of illness and to generate models for the association of signs and symptoms during the early phase of illness and over the entire course of illness with testing dengue-positive. In order to account for the longitudinal structure of the data, odds ratios (ORs) and 95% confidence intervals were calculated using generalized estimating equations (GEE), adjusting for age and gender.

## Methods

### Study site and participants

In August and September 2004, a community-based pediatric cohort was established in District II of Managua, a low-to-middle income area with a population of approximately 62,500 [21]. Study activity was based in the Health Center Sócrates Flores Vivas (HCSFV), a public facility that is the primary source of health care for District II residents. Briefly, participants aged 2–9 years were recruited through house-to-house visits, and additional two year-olds were enrolled each year to maintain the age structure of the cohort [21]. Children were eligible to remain in the study until age 12 or until they moved from the study area. The parent/legal guardian of each participant signed an informed consent form, and children ≥6 years old provided verbal assent. In 2007, participants ≤11 years old were given the opportunity to continue for an additional 3 years, and a second informed consent was performed.

### Ethics statement

The study was approved by the Institutional Review Boards of the University of California, Berkeley, the Nicaraguan Ministry of Health, and the International Vaccine Institute in Seoul, Korea. Parents or legal guardians of all subjects in both studies provided written informed consent, and subjects 6 years of age and older provided assent.

### Data collection

**Figure 1:**
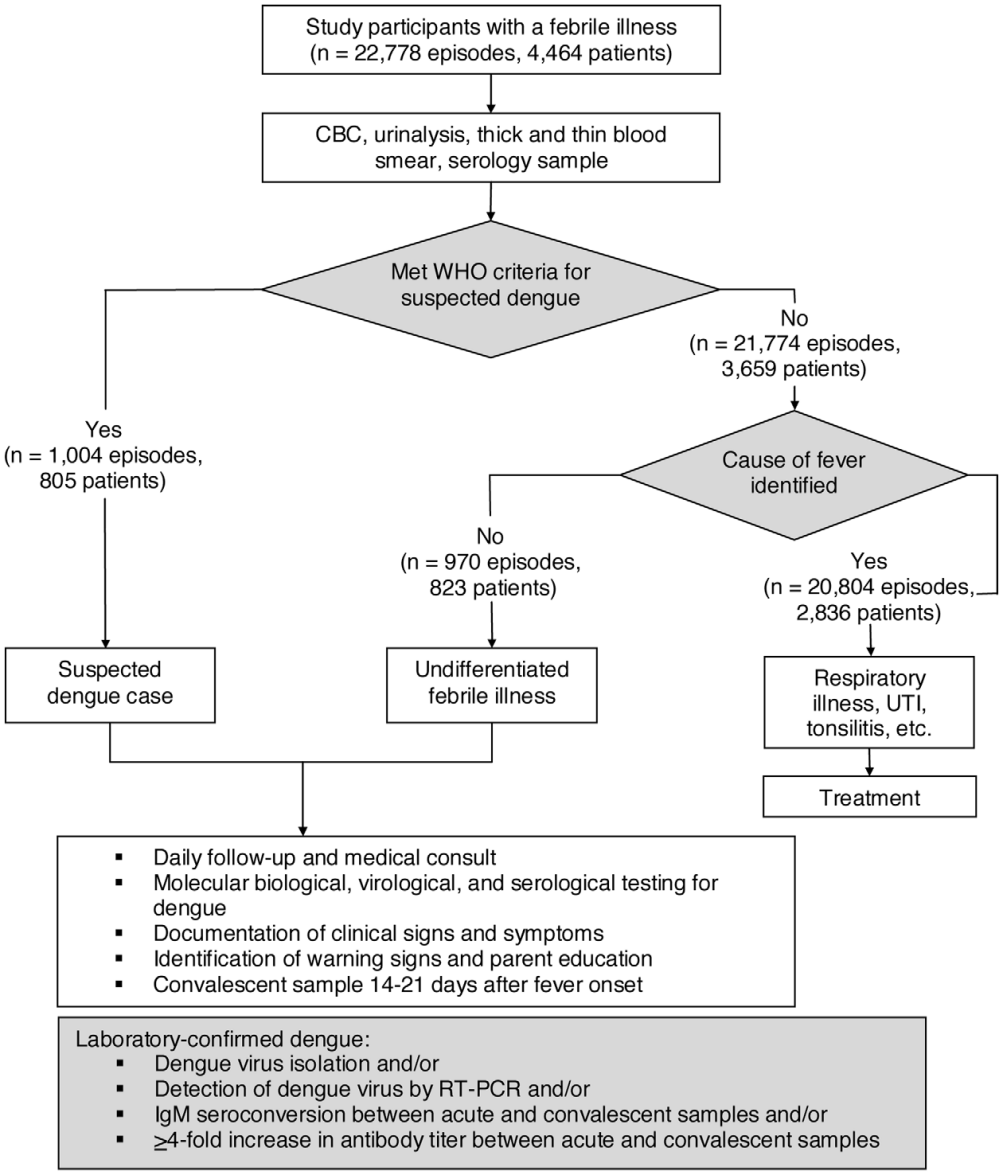
Flowchart of clinical and laboratory protocols for study participants in the Pediatric Dengue Cohort Study. Of the 1,974 episodes of febrile illness in the Pediatric Dengue Cohort Study from August 2004 to June 2009 that met the WHO classification criteria for suspected dengue or were diagnosed with undifferentiated fever, 405 patients presented with febrile illness on 2 occasions, 105 presented on 3 occasions, 21 presented on 4 occasions, and 5 presented on 5 occasions. One patient presented after day 8 of illness and was excluded from analysis. Twenty-nine patients had cause of fever identified later in the course of illness. CBC, complete blood count; WHO, World Health Organization; UTI, urinary tract infection.

Upon enrollment, parents/legal guardians of all participants were encouraged to bring their child(ren) to the HCSFV at first sign of illness or fever. Study physicians and nurses, trained in identification of possible dengue cases, provided medical care for study participants. Febrile illnesses that met the WHO criteria for suspected dengue (Table 1) and those without other apparent origin (undifferentiated febrile illnesses) were treated as possible dengue cases and followed daily while fever or symptoms persisted through visits with study medical personnel (Figure 1). Complete blood counts (CBCs) were completed every 48 hours or more frequently as necessary, as indicated by the physician. Cases were monitored closely for severe manifestations and were transferred by study personnel to the Infectious Disease Ward of the Manuel de Jesús Rivera Children's Hospital, the national pediatric reference hospital, when they presented with any sign of alarm (Table 1). In addition, an annual healthy blood sample was collected to identify all DENV infections during the previous year and for baseline CBC values. Study physicians in both the hospital and HCSFV completed systematic data collection forms that contained approximately 80 variables (Table 1). In the hospital, additional clinical data, including fluid balance and treatment, were collected daily during hospitalization or through ambulatory follow-up visits by a team of study physicians and nurses. Data were also recorded on medical tests ordered and treatments prescribed.

**Table 1:** Definitions of clinical terminology, variables and disease classifications.

		Definition
Term	Signs of alarm	Persistent vomiting, moderate to severe hemorrhagic manifestations, neurological manifestations, platelet count ≤100,000 cells/mm^3^, hematocrit ≥20% of normal value for age and sex
	Variables collected in hospital and health center systematic forms	Temperature, blood pressure, cardiac and respiratory rates, lower and upper respiratory symptoms, gastrointestinal symptoms, indicators of dehydration, urinary tract symptoms, musculoskeletal pain, rashes and other skin abnormalities, hemorrhagic manifestations, nutritional status
Variable	Fever	>37.8°C
	Narrow pulse pressure	≤20 mmHg
	Poor capillary refill	>2 sec
	Hypotension	Systolic blood pressure <80 mmHg for children <5 years of age and <90 mmHg for children ≥5 years of age
	Leukopenia	WBC ≤5000 cells/mm^3^
	Increased hematocrit	20% increase in hematocrit (compared to the stabilized hematocrit at hospital discharge) or hematocrit 20% above normal for age and sex
Classification	Suspected dengue	Acute febrile illness with 2 or more of the following: headache; retro-orbital pain; myalgia; arthralgia; leukopenia (WBC ≤5000 cells/mm^3^); rash; hemorrhagic manifestations
	Dengue hemorrhagic fever (DHF)^a^	All of the following must be present:
		Fever or history of acute fever lasting 2–7 days
		Hemorrhagic manifestations (positive tourniquet test; petechiae, equimosis, purpura or bleeding from mucosa, gastrointestinal tract, injection sites or other locations; hematemesis; melena)
		Thrombocytopenia (≤100,000 platelets/mm^3^)
		Evidence of plasma leakage due to increased vascular permeability
	Dengue shock syndrome (DSS)^a^	DHF with hypotension for age or narrow pulse pressure (≤20 mmHg) plus one of the following: rapid and weak pulse; cold, clammy skin; restlessness; poor capillary refill (>2 sec)
	Dengue with signs associated with shock (DSAS)^a^	Hypotension for age or narrow pulse pressure (≤20 mmHg) plus one of the following: poor capillary refill (>2 sec); cold extremities; weak pulse
	Dengue with compensated shock (DFCS)^a^	DF with poor capillary refill (>2 sec) plus one of the following on the same day: cold extremities; weak pulse; tachycardia; tachypnea
	Severe dengue	DHF, DSS, DSAS or DFCS

a plus laboratory confirmation of current dengue virus infection.

### Dengue classification

A case was considered laboratory-confirmed dengue when acute DENV infection was demonstrated by: detection of DENV RNA by RT-PCR; isolation of DENV; seroconversion of DENV-specific IgM antibodies observed by MAC-ELISA in paired acute- and convalescent-phase samples; and/or a ≥4-fold increase in anti-DENV antibody titer measured using Inhibition ELISA [22]–[25] in paired acute and convalescent samples. DENV serotypes were identified by RT-PCR and/or virus isolation.

Laboratory-confirmed dengue cases were further classified by severity. DHF and DSS were defined according to the traditional WHO criteria (Table 1) [26]. Additional categories of severity were included for those cases presenting with shock without thrombocytopenia and/or hemoconcentration (dengue with signs associated with shock (DSAS)) [23] or dengue fever with compensated shock (DFCS) [27] (Table 1). Laboratory-confirmed cases were defined as primary DENV infections if acute-phase antibody titer, as measured by Inhibition ELISA, was <1∶10 or if convalescent phase antibody titer was <1∶2560, and as secondary infections if the acute titer was ≥1∶10 or convalescent titer was ≥1∶2560 [22]–[25].

### Data

Data from the first five years of the study (August 30, 2004–June 30, 2009) were used for analysis. The first three days after onset of fever were considered the early febrile phase of illness. Day of illness at presentation was determined by the date of fever onset, which was defined as the first day of illness as reported by the parent/guardian. Variable definitions are described in Table 1. Positive tourniquet test was examined using cut-offs of ≥10 petechiae/in^2^ and ≥20 petechiae/in^2^. Platelet count was dichotomized using a cut-off of ≤150,000 cells/mm^3^ to enable comparisons during days 1–3. Only data from days 1–8 of illness were included for analysis.

### Statistical analysis

The frequency of dengue testing results (laboratory-confirmed dengue-positive versus dengue-negative) and disease severity (DF versus severe dengue) was examined by year, demographics, serotype and immune response. The frequency of the WHO case definition for suspected dengue was examined by dengue testing results and age, and a chi-square test for trend was performed. The frequency of clinical signs and symptoms by day of illness and dengue severity was also examined using chi-square tests.

To examine the association between clinical signs and symptoms and the odds of testing dengue-positive versus dengue-negative, odds ratios (ORs) and 95% confidence intervals (CIs) were calculated using GEE models assuming an exchangeable correlation structure with robust standard errors to account for the correlations between repeated measures on the same patients over time. First, ORs were calculated using bivariable models that included only dengue testing results and each of the signs or symptoms. All signs and symptoms were then examined in multivariable models that adjusted for age and gender. Data from the first three days of illness and from all days of illness only were analyzed separately. Finally, for comparison, we used traditional logistic regression models to analyze the association between signs and symptoms and testing dengue-positive with data collapsed by illness episode to disregard repeated measures on the same patients, using the same model generation process as for the GEE models. All analyses were conducted using STATA 10 (StataCorp LP, College Station, TX).

## Results

**Figure 2:**
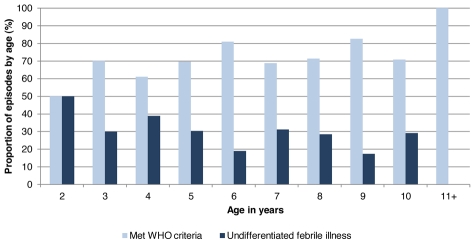
Frequency of dengue-positive episodes that met the WHO classification criteria for suspected dengue by age (n = 181). Upon presentation to the health center or hospital, children with a febrile illness were classified according to whether or not they met the WHO classification criteria for suspected dengue. One patient had two dengue virus infections over the course of the study and is represented twice. n = 6 for age 2, n = 10 for age 3, n = 18 for age 4, n = 23 for age 5, n = 21 for age 6, n = 16 for age 7, n = 21 for age 8, n = 23 for age 9, n = 24 for age 10, n = 19 for age 11+. Chi-square test for trend 5.977, p = 0.01. WHO, World Health Organization.

From August 2004 to June 2009, 22,778 episodes of febrile illness were evaluated, of which 1,974 episodes were suspected dengue or undifferentiated fever (Figure 1). Of the 1,974 possible dengue cases, 1,793 (91%) tested negative and 181 (9%) were laboratory-confirmed as dengue-positive, of which 161 (89%) were classified as DF, 9 (5%) as DHF, 4 (2%) as DSS, 3 (2%) as DSAS and 4 (2%) as DFCS (Table 1). Nearly all (95%) of the severe dengue cases but only 116 (72%) of the DF cases met the WHO case definition for dengue. The proportion of laboratory-confirmed DENV infections that met the WHO case definition significantly increased by age (chi-square test for trend 5.977, p = 0.01), while younger children experienced significantly more undifferentiated febrile illness due to DENV infection (Figure 2). The median age for cases meeting the dengue case definition was 8 years (range 2–13) and that of undifferentiated febrile illness due to DENV infection was 6 years (range 2–10).

The number of confirmed dengue-positive cases varied by year, as expected (Table 2) [28]. Both genders were equally represented, with a slightly higher percentage of females experiencing severe dengue, though this difference was not statistically significant. The majority of DF cases were DENV-2 (58%), followed by DENV-1 (21%) and DENV-3 (9%), while 60% of severe dengue cases were DENV-2, followed by DENV-3 (25%) and DENV-1 (10%). In addition, there were nearly equal proportions of primary and secondary immune responses among DF cases, whereas the majority (70%) of severe dengue cases were secondary DENV infection (Table 2). The median day of illness at presentation was day 2 for all patients, and almost all presented on days 1–3 of illness (90%). The total follow-up time of all children in the cohort was 17,931 person-years with a median follow-up of 3.9 years per child.

**Table 2:** Characteristics of study participants by dengue testing results and disease severity (n = 1,974).

		DENV-negative	DENV-positive	
		OFI(n = 1,793)	DF(n = 161)	Severe dengue(n = 20^a^)
		N (%)	N (%)	N (%)
Dengue season^b^	2004–05	312 (95)	16 (5)	1 (0)
	2005–06	516 (89)	63 (11)	2 (0)
	2006–07	397 (97)	12 (3)	1 (0)
	2007–08	328 (84)	53 (13)	11 (3)
	2008–09	240 (92)	17 (6)	5 (2)
Demographics	Female	864 (48)	75 (47)	11 (55)
	Male	929 (52)	86 (53)	9 (45)
	Median age in years (range)	6 (2–13)	7 (2–13)	9 (4–12)
	Median day of illness at presentation (range)	2 (1–8)	2 (1–8)	3.5 (1–6)
Serotype	DENV-1	N/A	33 (21)	2 (10)
	DENV-2	N/A	94 (58)	12 (60)
	DENV-3	N/A	14 (9)	5 (25)
	DENV-4	N/A	0 (0)	1 (5)
	Multiple	N/A	2 (1)^c^	0 (0)
	Indeterminate	N/A	18 (11)	0 (0)
Immune response	Primary	N/A	71 (44)	6 (30)
	Secondary	N/A	87 (54)	14 (70)
	Indeterminate	N/A	3 (2)	0 (0)

Numbers represent episodes of febrile illness. DENV, dengue virus; OFI, other febrile illness; DF, dengue fever; Severe dengue = dengue hemorrhagic fever (DHF), dengue shock syndrome (DSS), dengue with signs associated with shock (DSAS), or dengue fever with compensated shock (DFCS).

a Includes 9 DHF, 4 DSS, 3 DSAS, and 4 DFCS cases.

b Percentages are calculated horizontally for dengue season.

c Includes 1 case each of DENV-1/DENV-2 and DENV-1/DENV-4.

**Figure 3:**
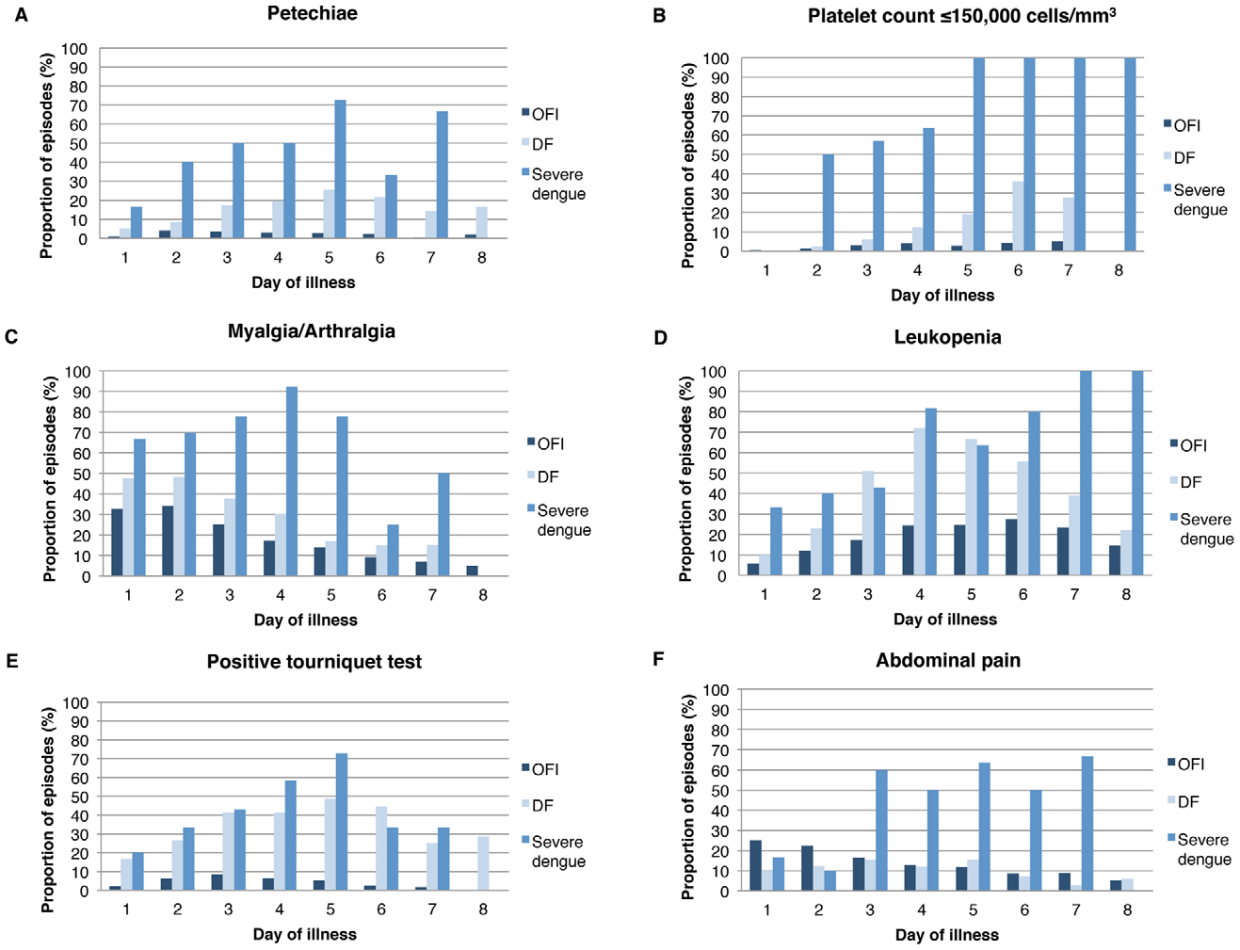
Frequency of signs and symptoms by day in patients with OFI, DF and severe dengue. Over the course of an episode of febrile illness, signs and symptoms were observed by medical personnel or reported by children and/or their parent/guardian. Selected signs and symptoms are shown here. A, Petechiae; OFI versus DF: chi-square test for trend 21.313, p<0.0001; day 1, n = 606; day 2, n = 1,243; day 3, n = 1,066; day 4, n = 876; day 5, n = 675; day 6, n = 481; day 7, n = 291; day 8, n = 175; B, Platelet count ≤150,000 cells/mm^3^; OFI versus DF: chi-square test for trend 14.928, p = 0.0001; day 1, n = 604; day 2, n = 970; day 3, n = 615; day 4, n = 568; day 5, n = 348; day 6, n = 234; day 7, n = 122; day 8, n = 65; C, Myalgia/arthralgia; OFI versus DF: chi-square test for trend 4.569, p = 0.03; day 1, n = 612; day 2, n = 1,253; day 3, n = 1,075; day 4, n = 877; day 5, n = 671; day 6, n = 477; day 7, n = 289; day 8, n = 181; D, Leukopenia; OFI versus DF: chi-square test for trend 6.449, p = 0.01; day 1, n = 604; day 2, n = 971; day 3, n = 615; day 4, n = 568; day 5, n = 348; day 6, n = 234; day 7, n = 122; day 8, n = 65; E, Positive tourniquet test; OFI versus DF: chi-square test for trend 20.124, p<0.0001; day 1, n = 256; day 2, n = 496; day 3, n = 402; day 4, n = 308; day 5, n = 202; day 6, n = 156; day 7, n = 78; day 8, n = 38; F, Abdominal pain; OFI versus DF: chi-square test for trend 9.149, p = 0.002; DF versus severe dengue: chi-square test for trend 4.127, p = 0.04; day 1, n = 609; day 2, n = 1,245; day 3, n = 1,066; day 4, n = 877; day 5, n = 675; day 6, n = 482; day 7, n = 290; day 8, n = 174; All other chi-square tests for trend comparing DF to severe dengue were non-significant. OFI, other febrile illness; DF, dengue fever; Severe dengue = dengue hemorrhagic fever, dengue shock syndrome, dengue with signs associated with shock, or dengue fever with compensated shock. Leukopenia is defined as WBC ≤5000 cells/mm^3^ and positive tourniquet test is defined as ≥10 petechiae/in^2^.

As shown in Figure 3, several signs and symptoms appeared to differentiate OFIs from DF cases, and DF cases from severe dengue cases, according to day of illness. In particular, higher proportions of DF and severe dengue cases experienced petechiae, platelets ≤150,000 cells/mm^3^, leukopenia, and positive tourniquet test compared to patients with OFIs. Higher proportions of severe cases experienced petechiae, platelets ≤150,000 cells/mm^3^, myalgia/arthralgia and abdominal pain compared to DF cases and patients with OFIs. Abdominal pain differentiated severe dengue cases from DF and OFI only beginning on day 3 of illness (for severe dengue compared to DF: chi-square 0.144, p = 0.70 for days 1–2 versus chi-square 16.910, p<0.0001 for day ≥3).

Bivariable and multivariable analyses were performed using GEE models to examine signs and symptoms early in illness and over the course of illness (Table 3). On days 1–3 of illness, dengue-positive cases had up to 2-fold increased odds of fever, headache, retro-orbital pain, myalgia, arthralgia, and vomiting compared to patients with OFIs. They also had from 3-fold to 9-fold increased odds of rash, petechiae, positive tourniquet test with cut-offs of ≥10 and ≥20 petechiae/in^2^, leukopenia, platelets ≤150,000 cells/mm^3^, poor capillary refill, cold extremities and hypotension compared to patients with OFIs. In contrast, they had decreased odds of abdominal pain, likely because this feature appears later in the entire course of dengue illness. On all days of illness, dengue-positive cases had increased odds of the same signs and symptoms as on days 1–3 of illness; however, the magnitude of the point estimates tended to be higher. This difference was most pronounced for rash and platelets ≤150,000 cells/mm^3^, which had ORs approximately double in magnitude. In addition, dengue-positive cases had increased odds of three additional signs and symptoms: poor appetite, absence of cough, and increased hematocrit. When GEE analyses on data with the longitudinal structure preserved were compared to traditional logistic regression analyses on data collapsed on febrile episode, the point estimates for the ORs were similar, although the 95% confidence intervals for the logistic regression models tended to be slightly narrower (data not shown).

**Table 3:** Signs and symptoms associated with testing DENV-positive among patients using generalized estimating equation models.

	Days 1–3		All days	
	OR (95% CI)	aOR (95% CI)^a^	OR (95% CI)	aOR (95% CI)^a^
Fever (>37.8°C)	1.7 (1.2–2.4)**	1.9 (1.3–2.7)***	1.8 (1.3–2.5)***	2.0 (1.4–2.7)***
Headache	2.0 (1.3–3.0)**	1.7 (1.1–2.7)*	2.0 (1.3–3.0)**	1.7 (1.1–2.6)*
Retro-orbital pain	1.8 (1.3–2.5)**	1.6 (1.2–2.3)**	2.2 (1.6–2.9)***	2.0 (1.4–2.7)***
Myalgia	2.0 (1.4–2.8)***	1.8 (1.3–2.6)***	2.4 (1.8–3.3)***	2.2 (1.7–3.1)***
Arthralgia	2.2 (1.6–3.0)***	2.0 (1.5–2.8)***	2.5 (1.9–3.5)***	2.4 (1.7–3.2)***
Rash	6.4 (4.0–10.2)***	6.6 (4.1–10.6)***	12.3 (8.4–18.0)***	12.5 (8.5–18.5)***
Petechiae	5.1 (3.2–8.3)***	5.1 (3.2–8.1)***	7.9 (5.3–11.8)***	7.8 (5.3–11.6)***
Positive tourniquet test (≥10 petechiae/in^2^)	9.3 (5.6–15.6)***	9.1 (5.4–15.3)***	13.5 (8.2–22.1)***	13.3 (8.1–21.8)***
Positive tourniquet test (≥20 petechiae/in^2^)	3.4 (2.4–4.9)***	3.3 (2.3–4.7)***	5.0 (3.7–6.9)***	4.9 (3.6–6.7)***
Abdominal pain	0.6 (0.4–0.9)**	0.6 (0.4–0.9)**	0.9 (0.6–1.3)	0.9 (0.6–1.2)
Poor appetite	1.4 (0.9–2.1)	1.5 (1.0–2.3)	2.0 (1.3–3.1)**	2.1 (1.4–3.3)**
Nausea	1.1 (0.6–1.9)	1.0 (0.6–1.8)	1.3 (0.8–2.1)	1.2 (0.7–2.0)
Vomiting	2.4 (1.6–3.6)***	2.4 (1.6–3.6)***	1.2 (1.1–1.3)**	1.2 (1.1–1.4)**
Sore throat erythema	1.2 (0.8–1.6)	1.1 (0.8–1.6)	1.2 (0.9–1.6)	1.2 (0.8–1.6)
Absence of cough	1.4 (0.8–2.6)	1.4 (0.8–2.5)	2.2 (1.0–4.6)*	2.2 (1.0–4.6)*
Leukopenia	4.7 (3.3–6.6)***	4.4 (3.1–6.4)***	7.6 (5.5–10.6)***	7.3 (5.3–10.1)***
Platelet count ≤150,000 cells/mm^3^	5.3 (2.6–10.7)***	5.2 (2.5–10.6)***	12.6 (7.9–20.1)***	11.9 (7.4–19.0)***
Increased hematocrit	1.4 (0.6–3.4)	1.2 (0.5–2.9)	2.7 (1.5–4.7)***	2.2 (1.2–3.9)**
Poor capillary refill	4.1 (1.3–13.3)*	4.7 (1.5–14.6)**	4.6 (1.6–13.3)**	5.1 (1.8–14.1)**
Cold extremities	6.2 (1.4–26.3)*	5.5 (1.4–21.8)*	4.8 (1.9–11.9)**	4.2 (1.8–10.0)**
Hypotension	2.8 (1.4–5.4)**	3.1 (1.6–6.0)**	2.6 (1.5–4.4)***	2.7 (1.6–4.6)***
Narrow pulse pressure	0.9 (0.5–1.5)	0.9 (0.5–1.5)	1.2 (0.8–1.7)	1.2 (0.8–1.7)

Generalized estimating equation models assume an exchangeable correlation structure with robust standard errors. DENV, dengue virus; OR, odds ratio; CI, confidence interval; aOR, adjusted odds ratio.

a ORs are adjusted for age and gender.

*p<0.05;

**p<0.01;

***p<0.001.

## Discussion

In this study, we describe the clinical spectrum of pediatric dengue starting early in illness in a community setting. Longitudinal statistical analysis of day-by-day clinical signs and symptoms revealed significant associations with testing dengue-positive and important differences during the early phase of illness compared to the entire course of illness. These results stress the importance of considering day of illness when developing prediction algorithms for real-time clinical management.

The early identification of dengue cases and particularly those at risk for severe dengue is critical for preventing severe illness and death. We found that 25% of laboratory-confirmed dengue cases did not meet the WHO case definition, suggesting that the WHO criteria are not sufficient to identify dengue at younger ages. Younger children may experience different signs and symptoms from adults or may be unable to communicate their symptoms to their parents, health care providers, or both. Previous studies demonstrated that children may experience significantly more cough, vomiting, abdominal pain, rash, epistaxis, oliguria, thrombocytopenia, hepatomegaly, and shock compared to adults, although the direction of these differences was not consistent across studies [13], [15], [29]–[34]. A recent study of dengue in adults showed significant differences in clinical features and outcomes across ten-year age groups, indicating that signs and symptoms associated with DENV infection may continue to evolve past childhood [12]. If these differences are confirmed, the WHO case definition may need to be adjusted to be age-specific to function effectively for younger children and older age groups.

Retro-orbital pain and low platelets were among the clinical features independently associated with DENV infection in this study. These results are supported by a study of dengue patients in Puerto Rico in which data were recorded at the time of initial consult rather than at hospitalization [15], and by a study of Thai children [11]. Moreover, our results showing increased frequency of abdominal pain in patients beginning at day 3 of illness are consistent with a prospective study of adults admitted to an emergency department in Martinique [35]. A positive tourniquet test using cut-offs of ≥10 and ≥20 petechiae/in^2^ was also independently associated with DENV infection. Both cut-offs were used because studies have indicated that a cut-off of ≥10 may improve discrimination of DENV infection [20], [36]; however, the 1997 WHO classification scheme specified a cut-off of ≥20 [26]. Our results support using a cut-off of ≥10 petechiae/in^2^, and this cut-off has been specified in the 2011 WHO clinical guidelines [37].

A major strength of this study is the use of statistical models designed for analysis of longitudinal data. Few other prospective community-based cohort studies have analyzed early clinical features in pediatric dengue compared to OFI [20], [38]–[40], and none that we are aware of were analyzed using longitudinal statistical methods that account for correlations between repeated measures on patients. Here, we preserved the longitudinal structure of the dataset by using statistical models that support repeated measurements on subjects over time and account for correlations between signs and symptoms experienced within the same individual on different days of illness and in multiple episodes. Longitudinal data have long been collected in dengue research but have rarely been analyzed using appropriate statistical methods. This may introduce bias into findings, as studies may overestimate the magnitude of association or reduce the statistical power of the study as data are lost when they are collapsed for non-longitudinal analysis.

An additional strength of this study is that it is community-based [21], enabling day-by-day capture of information on the early course of illness and on the full clinical spectrum of symptomatic dengue. In contrast, nearly all previous studies enrolled patients upon presentation to a hospital [18], where patients present later; thus, these studies were unable to capture information on the early days of illness or on mild disease. By examining the clinical spectrum of dengue by day of illness, we were able to detect differences in the prevalence of signs and symptoms that could not be revealed by simply analyzing whether they ever occurred over the course of illness. In addition, through multivariable longitudinal models, we were able to identify distinguishing features of dengue during the early phase of illness compared to the entire course of illness. These findings are important for clinical practice since outside of the hospital setting, clinicians may see dengue patients toward the beginning of their illness and utilize that information to decide whether their patient has dengue or another febrile illness. The results of these models should be extended for the development of prediction algorithms to aid clinicians in diagnosing suspected dengue.

This study was not without its limitations. Some participants migrated out of the study area or withdrew from the study; however, our retention rate was approximately 95% per year [21], suggesting that any bias from loss to follow-up would be minimal. It is also possible that we did not capture all symptomatic dengue cases. However, in yearly participant surveys, only an average of 2–3% of participants reported having attended a health-care provider outside of the study or having an illness and not attending any medical provider [21], and approximately 20-fold more laboratory-confirmed dengue cases were captured in the cohort study than by the National Surveillance System [41]. Unfortunately, due to the low number of severe dengue cases, this study did not have sufficient statistical power to compare severe dengue cases to DF cases using GEE models, and these low numbers may have influenced the lack of significant association of signs of severe dengue with testing dengue-positive. For this study, we used the 1997 WHO classification scheme for disease severity. In 2009, the WHO updated its guidelines for classification of dengue disease severity [1], [37]; it would be interesting to re-analyze the data in a future study using the new classification scheme. Studies of the usefulness and applicability of the revised guidelines have been recently performed [42], [43].

In summary, this study is one of the few cohort studies to provide early data on the full clinical spectrum of pediatric dengue. Though we found significantly increased odds for association of several clinical signs and symptoms with testing dengue-positive, these increases were more modest for the early phase of illness compared to the course of illness, suggesting that caution should be taken when using the results from the entire course of illness to develop prediction algorithms. Non-parametric methods such as decision tree analysis overcome some of the limitations of traditional logistic regression models and have recently been applied to develop algorithms for prediction of dengue diagnosis and disease severity [9], [44], [45]. These and other data-adaptive approaches such as Super Learner [46] that are less subject to bias should be further explored to develop prediction algorithms for early identification of dengue cases and improved clinical management.

## Supporting Information

Checklist S1 **STROBE checklist for cohort studies.**
(DOC)
